# Superelastic Radiative Cooling Metafabric for Comfortable Epidermal Electrophysiological Monitoring

**DOI:** 10.1007/s40820-023-01156-9

**Published:** 2023-07-13

**Authors:** Jiancheng Dong, Yidong Peng, Yiting Zhang, Yujia Chai, Jiayan Long, Yuxi Zhang, Yan Zhao, Yunpeng Huang, Tianxi Liu

**Affiliations:** 1 Key Laboratory of Synthetic and Biological Colloids, Ministry of Education, School of Chemical and Material Engineering, Jiangnan University, Wuxi, 214122 People’s Republic of China; 2 College of Energy Material and Chemistry, College of Chemistry and Chemical Engineering, Inner Mongolia University, Hohhot, 010021 People’s Republic of China

**Keywords:** Passive radiative cooling, Human electrophysiological monitoring, Superelastic metafabrics, Spectrally selective reflecting microfibers, Liquid metals

## Abstract

Epidermal electronics with superb passive-cooling capabilities are of great value for both daytime outdoor dressing comfort and low-carbon economy. Herein, a multifunctional and skin-attachable electronic is rationally developed on a porous all-elastomer metafabric for efficient passive daytime radiative cooling (PDRC) and human electrophysiological monitoring. The cooling characteristics are realized through the homogeneous impregnation of polytetrafluoroethylene microparticles in the styrene–ethylene–butylene–styrene fibers, and the rational regulation of microporosity in SEBS/PTFE metafabrics, thus synergistically backscatter ultraviolet–visible–near-infrared light (maximum reflectance over 98.0%) to minimize heat absorption while efficiently emit human-body midinfrared radiation to the sky. As a result, the developed PDRC metafabric achieves approximately 17 °C cooling effects in an outdoor daytime environment and completely retains its passive cooling performance even under 50% stretching. Further, high-fidelity electrophysiological monitoring capability is also implemented in the breathable and skin-conformal metafabric through liquid metal printing, enabling the accurate acquisition of human electrocardiograph, surface electromyogram, and electroencephalograph signals for comfortable and lengthy health regulation. Hence, the fabricated superelastic PDRC metafabric opens a new avenue for the development of body-comfortable electronics and low-carbon wearing technologies.

**Supplementary Information:**

The online version contains supplementary material available at 10.1007/s40820-023-01156-9.

## Highlights

- Efficient sunlight reflectivity and high mid-infrared radiation emissivity are simultaneously realized in a nonwoven metafabric via PTFE microparticle impregnation and thermal-fusion.
- The metafabric achieves a maximum cooling effect of 17 °C and fully retains its passive cooling performance even under 50% stretching.
- High-quality electrophysiological monitoring of ECG, sEMG and EEG is realized through compact and homogeneous encapsulation of liquid metal on the elastomeric fibers.

**Supplementary Information:**

The online version contains supplementary material available at 10.1007/s40820-023-01156-9.

## Graphical abstract

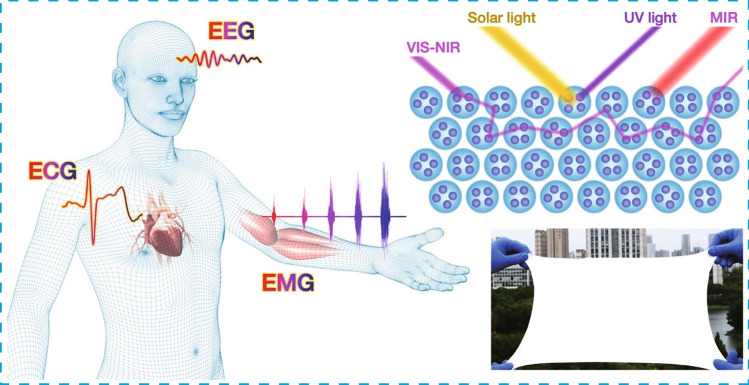

## Introduction

Wearable electronics show broad applications in point-of-care health regulation [1], human–machine interfaces [2, 3], and metaverse interfacing [4]. To accomplish their full potential for daily wearing comfort and lengthy serving reliability, researchers endeavor to explore the novel combinations of flexible substrates and deformable conducting materials featuring superior mechanical pliability, sufficient air/moisture permeability, as well as excellent mechano–electro response stability [5–10]. However, heat accumulation in the device-skin interface during long-term mounting may not only results in unstable signal acquisition, but can also cause discomfort and skin allergy [11, 12]. Fortunately, passive daytime radiative cooling (PDRC) materials can reflect daytime sunlight and synchronously emit thermal radiation from the human body to deep space (especially in the 8–13 μm waveband) [13, 14], providing fire-new opportunities for wearable devices with low-carbon cooling performance [15, 16]. For example, polyvinylidene fluoride (PVDF) nanomesh (~ 600 nm in diameter) with more than 90% UV–visible–NIR reflectance and ~ 50% IR transmittance was applied as PDRC textile, which presented a great cooling effect of 12 °C [17]. Other studies indicated that embedding TiO_2_ or ZnO nanoparticles into the yarns can effectively reflect solar radiation and selectively transmit the human body’s thermal radiation, also achieving a great cooling effect under daylight conditions [18, 19]. Nonetheless, the infrared emissivity of recently reported PDRC substrates created for wearable applications can hardly exceed 0.9 [20]. Worse still, the above-mentioned metal oxides may generate abundant free radicals due to the excessive absorption of UV and NIR radiation when reflecting sunlight, resulting in self-degradation, cooling efficiency decreasing, and even hazardous effects on human skin [21–23]. Apart from these, superelastic epidermal electronics with passive radiative cooling functionalities have rarely been reported.

Herein, multifunctional metafabrics with outstanding passive cooling and electrophysiological monitoring performance were fabricated on an ultrastretchable and highly breathable fibrous substrate. Polytetrafluoroethylene (PTFE) microparticles with inherently optical phonon resonances in the infrared wavebands but negligible absorption in the solar-radiated wavelengths were mixed with styrene–ethylene–butylene–styrene (SEBS) dope, which was then processed into elastic microfibers through facile electrospinning. Facilitated by the thermoplastic property and high thermal stability of SEBS, obtained nonwoven fabrics were treated by thermal fusion to regulate the pore size and reinforce the mechanical strength. Lastly, liquid metal (LM) was eventually printed onto the metafabric and was activated through pre-stretching to endow the fabric with stable and deformable conductivity. Fabricated metafabrics showed a remarkable water vapor permeability of 2495.9 g m^−2^ day^−1^ and an ultra-high stretchability of up to 1177.6%. Taking advantage of the evenly embedded PTFE microparticles and the thermal-regulated micropores with tailored pore size, efficient sunlight reflecting performance and high mid-infrared radiation emissivity were simultaneously achieved, realizing a maximum cooling performance of 17 °C under both cloudless and cloudy outdoor daytime atmosphere conditions, and excellent cooling effect even under 50% stretching. Particularly, the PDRC metafabrics applied as on-skin sensory devices showed a comparably high sensing performance as commercial gel electrodes for human physiological signals, including human electrocardiograph (ECG), surface electromyogram (sEMG), and electroencephalograph (EEG) signals.

## Experimental Section

### Materials

SEBS was obtained from Kraton Co., Ltd. PTFE were purchased from Dongyang Polymer Materials Co., Ltd. Gallium–indium eutectic (EGaIn, Ga/In = 75.5%/24.5%) was supplied by Dongguan Huatai Metal Material Technology Co., Ltd. 1-Ethyl-3-methylimidazolium bis(trifluoromethyl sulfonyl) imide (IL) and laboratory solvents were all supplied by Sinopharm Co., Ltd.

### Fabrication of the Nonwoven PDRC Metafabric

Firstly, a homogenously mixed solvent of chloroform and toluene (mass ratio: 9/1) was prepared, and then, a few drops of IL were instilled to increase the electric conductivity of the solution. After that, the SEBS masterbatch was dissolved in the above solvent under stirring to obtain the SEBS dope with a mass concentration of 15 wt%, and then, an appropriate amount of PTFE powders was added into the SEBS solution under vigorously stirring to yield the electrospinning dope. The dope was transferred to a syringe for electrospinning in a sealed chamber, and the obtained nonwoven fabrics (denoted as SPM) were received onto a copper mesh to enable permeability. The detailed spinning parameters were the same as in our previous works [24].

The produced SEBS/PTFE microfiber fabrics were thermal-treated at 150 °C for different times before peeling off from the copper mesh, which were then washed with ethanol and dried at the ambient environment. (Obtained samples were denoted as tSPM). Lastly, EGaIn was painted onto one side of the tSPM fabric using a soft multi-headed brush, followed by activation through cyclic monolithic stretching to obtain the passive-cooling metafabric.

### Materials Characterizations

Scanning electron microscopy (SEM) images were taken with a Hitachi S-4800 FE-SEM. Optical microscopic images were captured by the Keyence VHX-1000C Ultra Depth Microscope. 3D morphologies were recorded using a Keyence VK-X150 Laser Microscope. X-ray diffraction (XRD) patterns were recorded via a Bruker AXS D2 PHASER X-ray diffractometer. Attenuated total reflection Flourier transformed infrared spectroscopy (ATR-FTIR) spectra were measured with the Thermofisher Nicolet iS50 FT-IR. The reflectance (*R*) and transmittance (*T*) of samples were measured using a Shimadzu UV-3600 plus spectrometer in the range of 0.25–2.5 µm with an integrating sphere, and a Thermofisher Nicolet iS50 FT-IR spectrometer coupled with a gold integrating sphere was utilized in the range of 2.5–25 µm. The emittance **e** was calculated through *E* (%) = 100% − *R*(%) − *T*(%).

Water moisture permeability of the fabric was characterized with a W3/060 Water Vapor Transmission Rate Test System (temperature: 30–40 °C, humidity: 80 RH%). Mechanical properties were measured via a Suns Technology universal UTM2203 tensile testing machine. Electromechanical properties were measured utilizing the Keysight 34465A digital meter coupled with a Mark-10 ESM303 tensile tester.

### Monitoring of ECG, sEMG, and EEG Signals

The metafabric was cut into circular shapes to assemble the bioelectrodes for biosignal measurements. Then, three electrodes were mounted to the clavicles (measure electrodes) and iliac crest (reference electrode) of a male volunteer with an ECG plugin. Collected ECG signals were transferred to a cell phone or computer via the Bluetooth module, which were converted through Eq. (1) (valid ECG sensor range: − 1.5 to + 1.5 mV):

1 $${\text{ECG}} \left( {{\text{mV}}} \right) = \frac{{\left( {\frac{{{\text{ADC}}}}{{2^{n} }} - \frac{1}{2}} \right) \times {\text{VCC}} \times 10^{3} }}{{G_{{{\text{ECG}}}} }}$$

where ECG (mV) is the ECG signal in milliVolt (mV), VCC is the operating voltage (3.3 V), *G*_ECG_ is the sensor gain (1100), ADC is the collected digital value from the corresponding channel, and *n* is the number of bits of the channel (10 bits in this work).

The recorded sEMG signals were also processed through a conversion formula in Eq. (2) (valid sEMG sensor range − 1.64 to + 1.64 mV):

2 $${\text{sEMG}} \left( {{\text{mV}}} \right) = \frac{{\left( {\frac{{{\text{ADC}}}}{{2^{n} }} - \frac{1}{2}} \right) \times {\text{VCC}} \times 10^{3} }}{{G_{{{\text{sEMG}}}} }}$$

where sEMG (mV) is the sEMG signal in milli-Volt (mV), VCC is the operating voltage (3.3 V), *G*_sEMG_ is the sensor gain (1009), ADC is the collected digital value from the corresponding channel, and *n* is sampling resolution (10 bits in the research).

The EEG signals were further captured and converted through the following Eq. (3) (valid EEG sensor range: − 39.49 to + 39.49 μV):

3 $$EEG \left( {\mu V} \right) = \frac{{\left( {\frac{ADC}{{2^{n} }} - \frac{1}{2}} \right) \times VCC \times 10^{6} }}{{G_{EEG} }}$$

where EEG (mV) is the EEG signal in microvolt (μV), VCC is the operating voltage (3.3 V), *G*_EEG_ is the sensor gain (41,782), ADC is the collected digital value from the corresponding channel, and *n* is the number of bits of the channel (10 bits in this work).

## Results and Discussion

### Design and Fabrication of the PDRC Metafabric

**Fig. 1:**
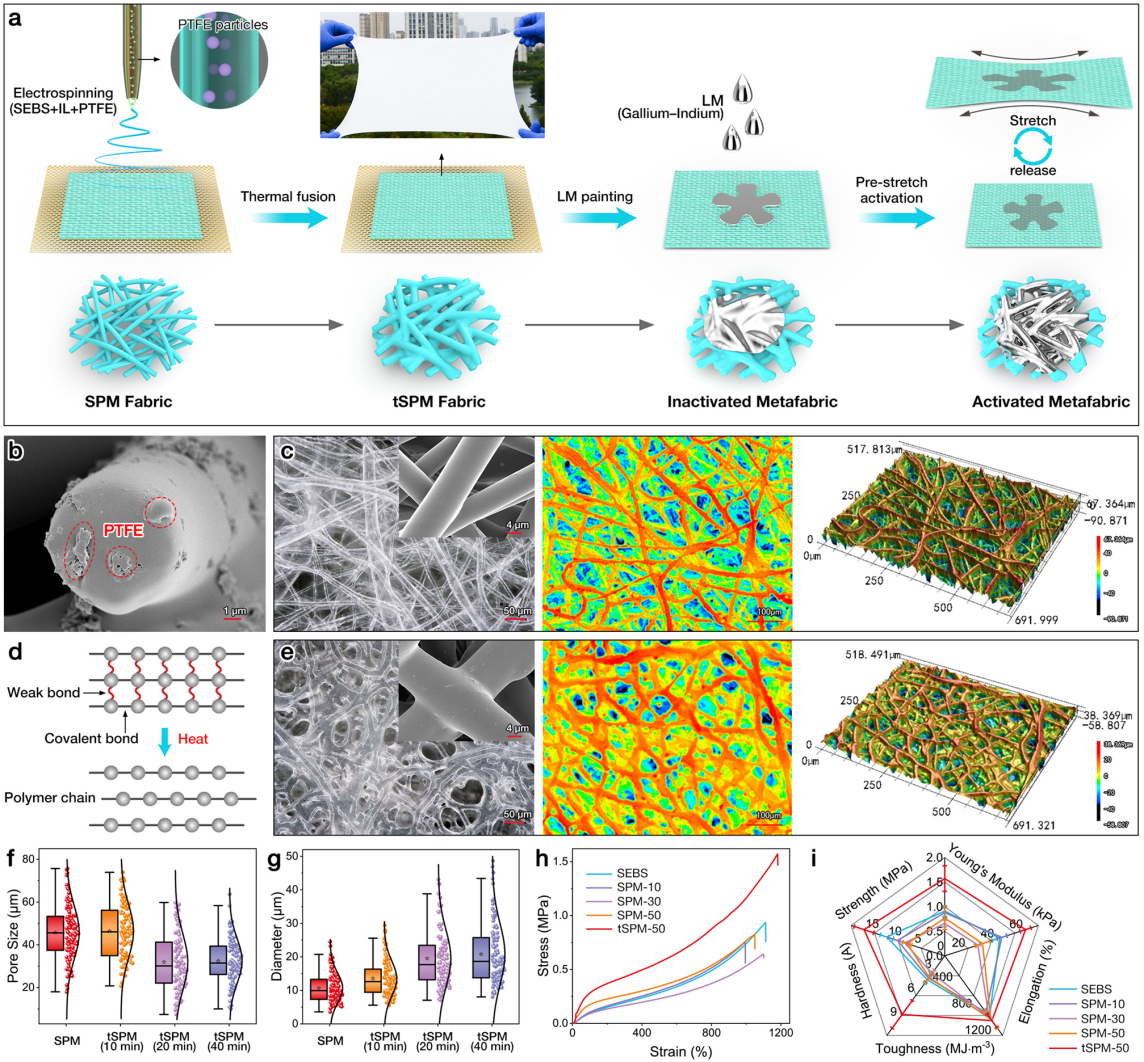
**a** Fabrication process of the PDRC metafabric. **b** Cross-sectional SEM image of SPM fiber. **c** Optical, contour and 3D images of the SPM microfibers (inset: amplified SEM image). **d** Thermoplasticity mechanism of SEBS. **e** Optical, contour and 3D images of tSPM microfibers (inset: amplified SEM image). **f, g** Pore size and diameter distribution of the SPM and tSPM fabrics treated for different times. **h** Strain–stress curves and **i** comprehensive mechanical properties of SEBS fabric, SPM fabrics with TPFE content of 10 ~ 50 wt%, and tSPM fabric containing 50 wt% PTFE

The application of on-skin electronics in practical scenarios must not only meet the requirements of conformability and adaptability between electronics and soft skin, but should also take skin physiological conditions into account. The passive cooling capability of skin devices provides a healthier microenvironment for human skin in hot outdoor conditions. For flexible and stretchable electronics, fully maintained PDRC performance under large-scale deformation is particularly beneficial for comfortable wearing under sports activities. In this research, an all-polymer and ultrastretchable PDRC metafabric consisting of spectrally selective reflecting microfibers and an infinitely deformable liquid metal circuit is developed for long-term comfortable and point-of-care monitoring of human electrophysiological signals. The microfibers with rationally regulated micropores can lead to strong Mie scattering effects, while the embedding of PTFE microparticles further enhances the scattering effects, realizing high sunlight reflection. Moreover, thermal treatment is also expected to reinforce the mechanical strength by fusing the interweaved elastic fibers, ensuring the robustness and stability of the electronics during long-term serving. The whole fabrication process of the PDRC metafabric is depicted in Fig. 1a.

Benefiting from the simple processing, a large-size nonwoven SPM fabric in 30 cm × 45 cm × 200 μm dimension can be readily produced, and it can be stretched easily along the four corners owing to the superior elasticity. As shown in Fig. S1, pure SEBS nonwoven consists of smooth and transparent micro-sized fibers, forming porous and interconnected fibrous networks. PTFE microparticles (200 ~ 1600 nm, Fig. S2) with low UV/NIR absorptivity yet high UV–visible–NIR reflectance were evenly embedded into the elastic microfibers to maximize the Mie scattering. Figures 1c and S3 present the optical and SEM images of SPM microfibers with varied PTFE content of 10–50 wt% (fibers with PTFE content beyond 50 wt% cannot be continuously produced). It could be observed that SPM microfibers became gradually opaque upon increasing the PTFE fraction: however, the porous fibrous structures were well maintained. The PTFE microparticles saturated the fiber matrix when the loading percentage reached 50 wt%, that a small part of the particles emerged on the fiber surface (Fig. 1b). More PTFE microparticle loading may lead to better sunlight reflective performance. The contour plot in Fig. 1c shows that SPM fibers (50 wt% PTFE) randomly distribute and overlap with each other, and formed multiscale pores are highly beneficial for the breathability of skin-attached electronics. Besides, FTIR peaks of SPM fibers at 1206, 1154, and 640 cm^−1^ are attributed to the –CF_2_ and –CF_3_ groups of PTFE [25], whereas the XRD diffraction peak at 18° also can be indexed to the (100) plane of PTFE (Fig. S4) [26], confirming the successful incorporation of PFTE microparticles in SEBS fibrous matrix.

As a well-known thermoplastic styrene copolymer, SEBS consists of hard polystyrene and soft polyethylene and polybutene, and therefore, it has two glass transition temperatures (*T*_g_) with one below subzero temperature (~ − 40 °C, caused by the soft phase) and the other close to the *T*_g_ of polystyrene (> 100 °C) [27–30]. Thermogravimetric analysis (TGA) curves of SEBS and SPM microfibers further proved the thermal stability of the elastomeric fabrics, wherein no obvious mass loss can be observed when rising the temperature up to 350 °C (Fig. S5). However, the polymer chains of SEBS are generally tangled together via weak interactions like Van der Waal’s forces and dipole–dipole attractions [31], such interactions can be deranged by thermal treatment and recovered after cooling (Fig. 1d) [27]. Taking advantage of the thermoplastic properties and high thermal stability of SEBS, a straightforward yet efficient thermal fusion strategy was used to regulate the pore size of the SPM fabric. As shown in Fig. 1e, adjacent fibers are thermal-fused together to form a more compact fibrous structure, with the fiber diameter increasing, while the pore size greatly decreases. More clearly, the contour plot of tSPM microfibers in Fig. 1e also verifies the fused fibers and the condensed porosity. Fig. S6 and S7 show the digital and optical images of tSPM fabrics treated at 150 °C for different times. Specifically, the pore size of the fabric changes from 45.8 to 46.39, 31.9, and 32.2 μm when the thermal treating time is 0, 10, 20, and 40 min, respectively (Fig. 1f). Accordingly, the fiber diameter increases from 11.5 to 13.4, 19.8, and 21.2 μm, respectively (Fig. 1g). Notably, long-time thermal treatment would inevitably cause the degradation and yellowing of the elastomer, and even worse, the porous fabric will gradually transform into impermeable film due to the closing of the voids (> 60 min, Fig. S8). Based on the above results, tSPM fabrics thermal-fused for 20 min were selected for the following applications.

Typically, SEBS shows rubber-like elasticity because the styrene-based hard blocks act as crosslink points below the *T*_g_ and the soft blocks provide elasticity [32]. As shown in Fig. 1h, all the strain–stress curves of prepared stretchable microfibers are S-shaped curves, in which the stiffness decreases with the load, and the fibers become stiffer and fracture at maximum stretchability. Among all the samples, tSPM fabric shows the best overall mechanical performance in terms of strength (1.6 ± 0.3 MPa), Young’s modulus (66.1 ± 3.7 kPa), elongation (1178 ± 100%), shore hardness (16. 7 ± 2.1 A), and toughness (8.4 ± 1.7 MJ m^−3^) (Fig. 1i). Whereas pure SEBS and all SPM samples before thermal treatment show comparatively lower strength than that of thermal-treated one (e.g., the strength of SPM-50 is as low as 0.8 ± 0.2 MPa). It is also notable that more PTFE content in the SEBS fibers may inevitably lead to mechanical degradation in the tSPM fabrics. For example, the Young’s modulus of pure SEBS microfibers decreases from 48.2 ± 5.1 to 36.2 ± 5.0 kPa of SPM-50, and its toughness also decreases from 11.7 ± 1.4 to 3.7 ± 0.6 MJ m^−3^. In addition, the softness (< 20 A) and Young’s modulus (below 100 kPa) of tSPM fabric are very close to those of the human skin (Fig. S9), manifesting the favorable characteristics of the metafabric in flexible and wearable devices.

**Fig. 2:**
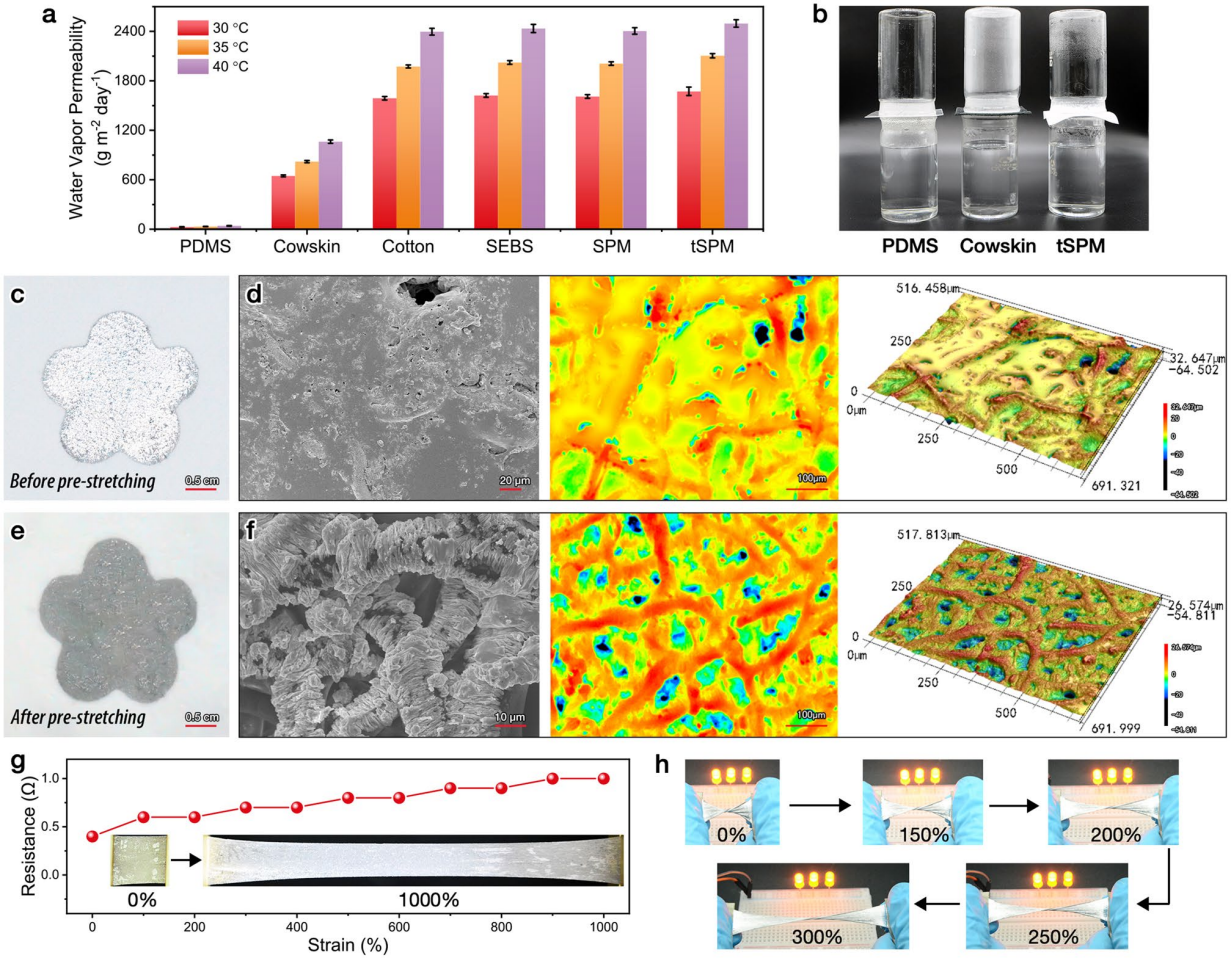
**a** Water vapor permeability of PDMS film, cowskin, cotton fabric, SEBS nonwoven fabric, SPM and tSPM fabrics. **b** An intuitive demonstration on the permeability of different samples. **c, e** Digital photos of flower-shaped LM printed on tSPM microfibers before and after pre-stretch activation, and **d, f** corresponding SEM, contour and 3D images of the metafabric. **g** Resistance of the metafabric when stretching up to 1000%. **h** Electrical conductivity of the metafabric under large-scale stretching and twisting

In addition to the reliable mechanical performance, adequate breathability of on-skin electronics is indispensable in keeping and adjusting the skin microenvironment. The comparison of aqueous vapor permeability under different temperatures between different fabrics is presented in Fig. 2a. It is no doubt that the permeabilities of all samples increase with the temperature rise. Pure SEBS, SPM, and tSPM fabrics exhibit comparable permeabilities as the porous cotton fabric, which can completely fulfill the daily sweating needs of the skin. Specifically, tSPM fabric shows the vapor permeabilities of 1673, 2105, and 2496 g m^−2^ day^−1^ at 30, 35, and 40 °C, respectively, over two times larger than that of commercial leather. On the contrary, hermetical PDMS film (~ 200 μm in thickness) presents nearly zero moisture permeability, which was broadly applied in elastic electronics and severely suffered from perspiration accumulation problems [33, 34]. To intuitively observe the breathability of the samples, different fabrics and films were placed between two vials, with the top one empty and the bottom one filled with hot water (Fig. 2b). Distinctly, the water vapor penetrated through the tSPM microfibers quickly and condensed inside the vial in minutes. Whereas the top vial remained dry or partially wetted when the tSPM fabric was replaced by the casted PDMS film or the cowskin, proving the excellent vapor moisture permeability of the tSPM fabric. Moreover, pure SEBS microfibers are intrinsically hydrophobic with a water contact angle (WCA) of 128.8 ± 4.4° (Fig. S10a). whereas, the tSPM fabric has a WCA up to 138.5 ± 1.3° (Fig. S10b) due to the embedding of superhydrophobic PTFE microparticles (WCA = 141.8 ± 3.8°, Fig. S10c), which also manifests remarkable waterproof performance even under the ultra-large strain of 1000% (Fig. S10d).

Lastly, an infinitely deformable EGaIn circuit was printed onto the tSPM fabric and then activated via pre-stretching. As shown in Fig. 2c, the flower-shaped circuit can be easily printed on the elastic nonwoven fabric to serve as a bioelectric sensor. However, instead of penetrating the fabric, the freshly printed EGaIn is completely floating on the surface of the fabric (Fig. 2d), potentially leading to the deterioration of air permeability. Figure 2e, f shows the digital and microscopic images of the activated LM circuit. The shiny silver appearance of the LM circuit turns grayish after a simple monolithic stretching, which can be ascribed to the oxidation of EGaIn as a result of the enlarged surface area, in accordance with previously reported works [35]. In particular, the EGaIn permeated into the tSPM fibers and transferred into the dense and homogeneous coating on single fibers (SEM image in Fig. 2f), forming wrinkled microstructures due to the mechanical competition between the harder upper gallium oxide layer and the softer underlying elastic fibers. Such special structures can help improve the intimate contact between the sensor and corrugated human skin [36, 37]. Surprisingly, the pre-stretching process also recovered the highly porous structure of the nonwoven fabric, as proved by the contour map and 3D mapping in Fig. 2f. The LM-printed metafabric shows remarkable electrical conductivity under large strains due to the combination of the ultraelastic SEBS substrates and the super-stretchable liquid metal. Figure 2g displays that the resistance of the metafabric merely increased from 0.5 to 1.0 Ω even under 1000% stretching. As a demonstration, a piece of conductive metafabric utilized as the elastic wire can light up an LED bulb under complex mechanical deformations including elongation, bending, and twisting (Fig. 2h).

### Passive Daytime Radiation Cooling Performance of the Metafabric

**Fig. 3:**
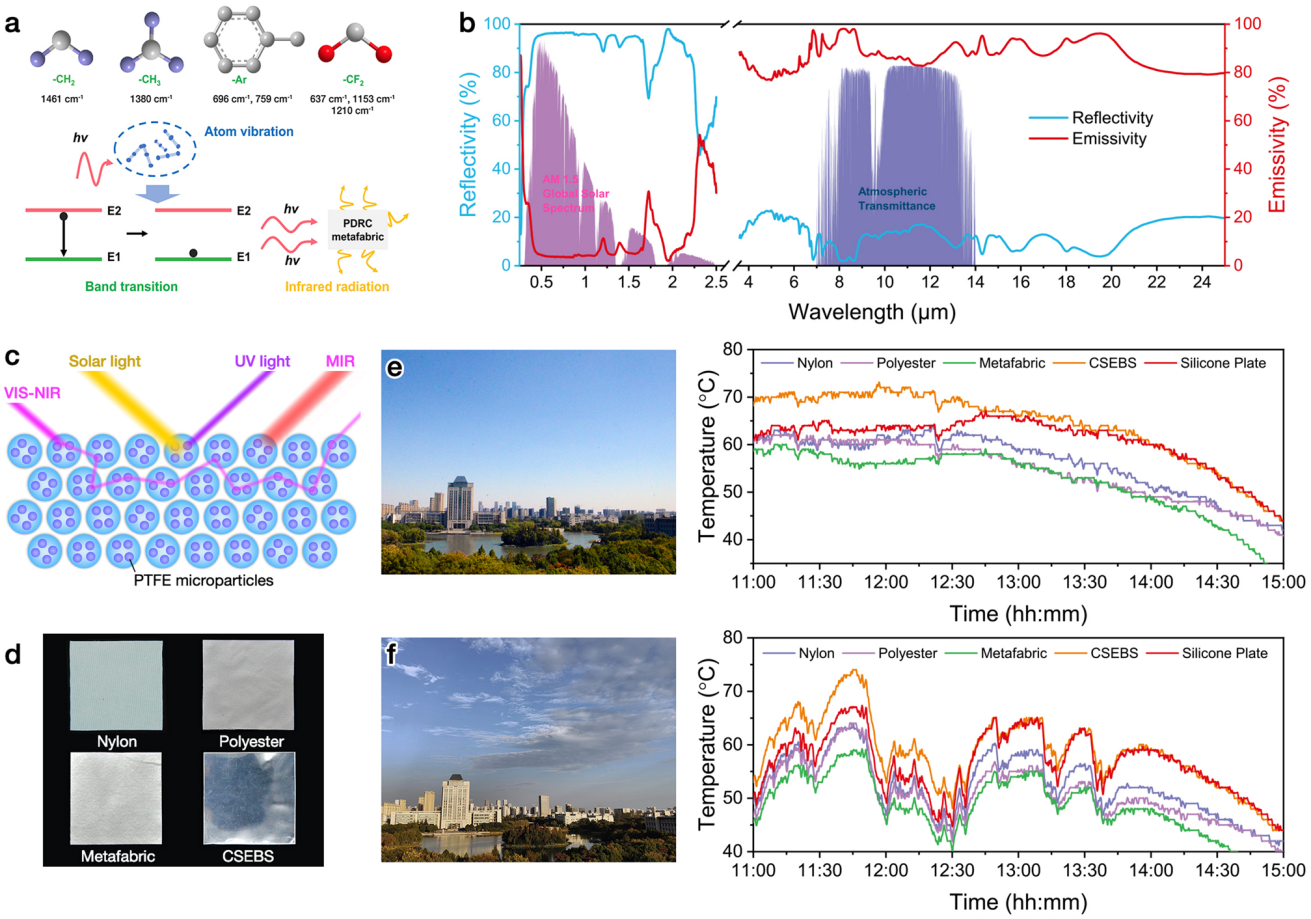
**a** Typical PDRC mechanisms of the fabricated metafabric. **b** Measured reflectivity and emissivity spectra of the metafabric (0.3 to 25 mm). **c** Schematic of the metafabric for radiative cooling. **d** Digital photos of the samples for the cooling tests. Continuous temperature measurements of the passive cooling performance in **e** clearly and cloudless sky and **f** cloudy sky conditions

Heat extremes (e.g., heatwaves) impose serious threats on human health, with aging and chronic illnesses as aggravating factors [38]. Via embedding high sunlight reflective yet low UV/NIR absorptive PTFE microparticles into the elastic microfibers, and regulating the pore size to realize the maximum UV–visible–NIR reflection in the nonwoven fabric, breathable and superelastic metafabrics with passive cooling capability were rationally developed for outdoor personal thermal management. Herein, at least three synergistic cooling mechanisms are involved in the PDRC metafabric. First of all, the SEBS/PTFE polymer matrix exhibits strong stretching or bending vibrations in the mid-infrared region (MIR) owing to specific chemical bonds including –CH_2_– (1461 cm^−1^), –CH_3_ (1380 cm^−1^), –CF_2_– (1210, 1153 and 637 cm^−1^), and –Ar (benzene ring, 759 and 696 cm^−1^) (Fig. 3a) [25, 39, 40]. As a result, the metafabric would receive radiation energy (hν) and accelerates molecular motion when exposed to sunlight irradiation, and then, the inner atoms will sustain drastic stretching vibrations. At the same time, the electrons will transition from the high-energy band to the low-energy band, leading to photon release and heat irradiation in the MIR region. Next, the PTFE particles embedded in the microfibers have a diameter range of 200–1600 nm (Fig. S2), which possess the collective effect of multiple Mie resonances and scattering peaks covering the whole UV–VIS–NIR band with high efficacy [19]. Lastly, the thermal-fused fiber networks (average pore size: 31.9 μm) can provide an expanded spectroscopic response that spans from 0.3 to 25 μm, thus empowering the metafabric with the capability to resonantly reject solar power and intensively emit body heat in the MIR band. Efficient emissivity in the full MIR band could be very advantageous for PDRC materials. Additionally, the fiber diameter of the metafabric (13.4 μm) is also very suitable for MIR emission [41], allowing for continuous body heat irradiation toward the outside.

Benefitting from the above structural and compositional advantages, the PDRC metafabric shows a wideband reflectivity of 92.4% in the solar radiation area (0.3–2.5 μm), and a mean emissivity of 94.5% throughout the atmospheric transparent spectral window (ATSW, 8–13 μm) (Fig. 3b). The wide band of high emissivity from 4 to 25 μm could provide extra cooling efficiency for the metafabric. The outdoor PDRC performance of the metafabric was directly measured under two typical weather conditions, namely clear sky and cloudy sky in Wuxi, China (31°28′55.8″ N, 120°16′37.5″ E). Figures 3c and S11 are the measurement setup and the possible cooling mechanism. Infrared transparent PE films were applied to seal the aluminum foil coated polystyrene (PS) box to eliminate the interference of thermal convection and conduction. Figure S12 shows that there was a large temperature difference between the air inside and outside the PS box, demonstrating the necessity of PE film. The samples (Fig. 3d) were directly placed onto a large piece of silicone Joule heater. The silicone plate can reach to a very high temperature when exposed to direct sunlight, which could be a suitable background for PDRC performance evaluation. The precise temperature change of every sample was recorded by a K-type thermocouple taped behind the samples. To start with, the metafabric showed much better cooling effectiveness than that of pure SEBS (Fig. S13). During a clear and cloudless day, the PDRC metafabric showed the lowest temperature among all the samples, presenting the maximum cooling effects of 17, 6, 5, and 8 °C with respect to CSEBS (cast impermeable SEBS film), nylon, polyester, and bare silicone plate in the peak solar irradiance (around 12 o’clock, Fig. 3e). Besides, the passive daytime radiative cooling performance under cloudy conditions was also measured to verify its weather versatility. Evidently, the metafabric also showed the best cooling performance when compared with other samples, demonstrating its broad application potential (Fig. 3f).

**Fig. 4:**
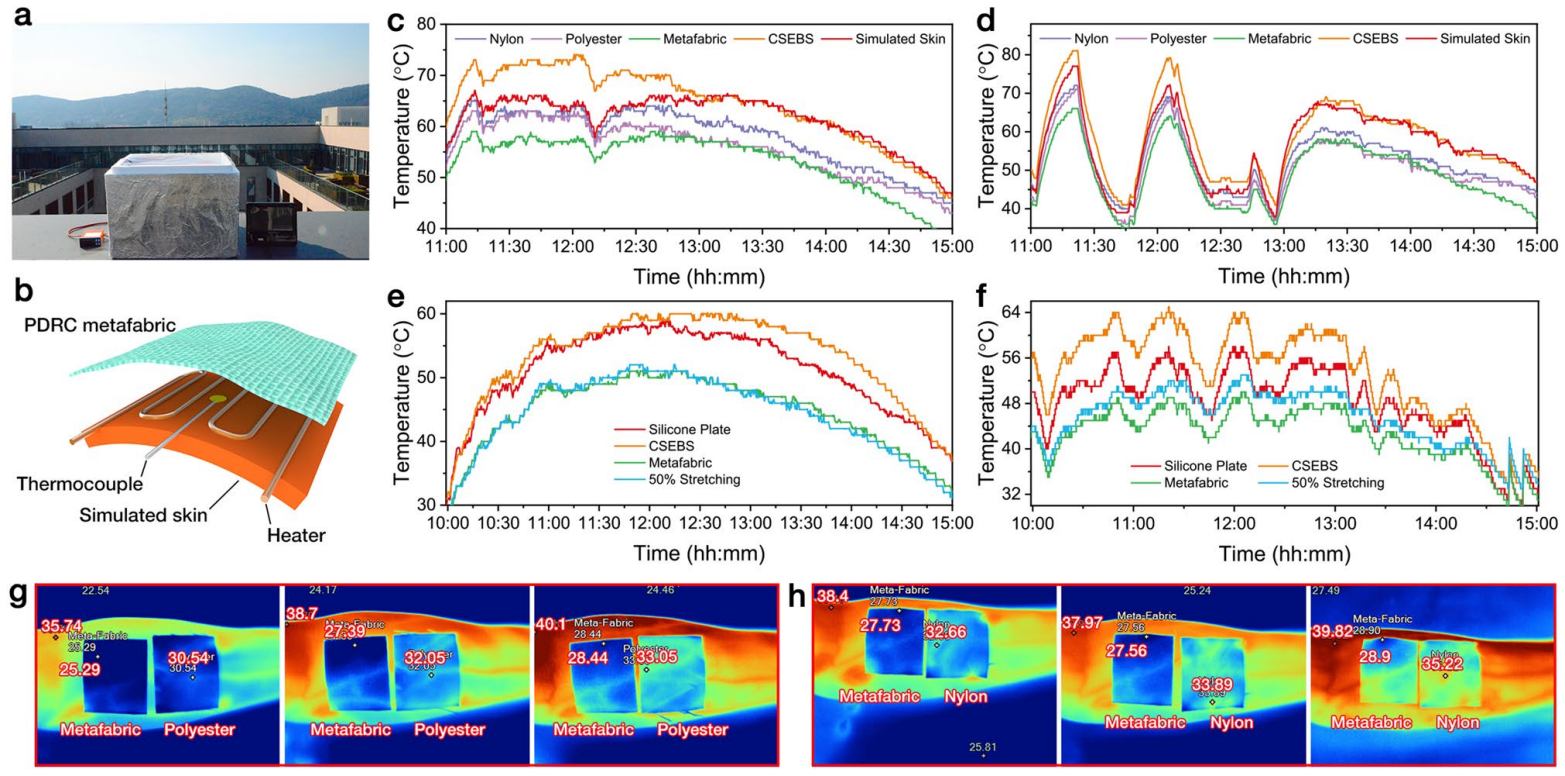
**a, b** Photograph and schematic of the device used for the skin simulator cooling tests. Temperature of the skin simulator under different fabric samples in **c** clear sky and **d** cloudy sky conditions. PDRC tests on the metafabric under 50% stretching in **e** clear sky and **f** cloudy sky. **g, h** Practical cooling tests on human skin using metafabric, nylon and polyester fabrics

Apart from the measurements in ambient environments, the cooling performance of the PDRC metafabric on a human skin simulator was also evaluated (Fig. 4a). Under peak solar irradiation from 11 to 15 o’clock, the temperature of the metafabric was about 17, 12, 10, and 7 °C lower than that of the CSEBS film, bare skin simulator, nylon fabric, and polyester fabric, no matter in a cloudless sky or upon cloudy condition (Fig. 4b, c). Considering the ultra-stretchability of the metafabric, its cooling performance was further investigated under stretching. Surprisingly, the metafabric fully maintained exceptional cooling performance even under 50% stretching at both the ambient environment (Fig. 4d) and human skin simulator (Fig. 4e) in cloudless sky conditions. Notably, the cooling curves of the stretched metafabric are almost in line with the pristine metafabric during the whole irradiation period (10:00 ~ 15:00 o’clock). The extremely stable PDRC performance of the metafabric can be ascribed to the robust fiber networks and the firmly embedded PTFE particles, which enables itself with great compatibility to serve under complex outdoor environments. In order to verify the cooling performance of the metafabric in practical situations, PDRC metafabric, nylon fabric, and polyester fabric were placed on the forearm of a volunteer for a direct comparison. A Fluker Ti 400 + thermal camera was applied to record the temperatures of different samples. Under direct sunlight irradiation for half an hour, the temperature on the PDRC metafabric was much lower than that on nylon and polyester fabrics (Fig. 4f, g), displaying its highly competitive cooling performance.

### Monitoring of Human Electrophysiological Signals

**Fig. 5:**
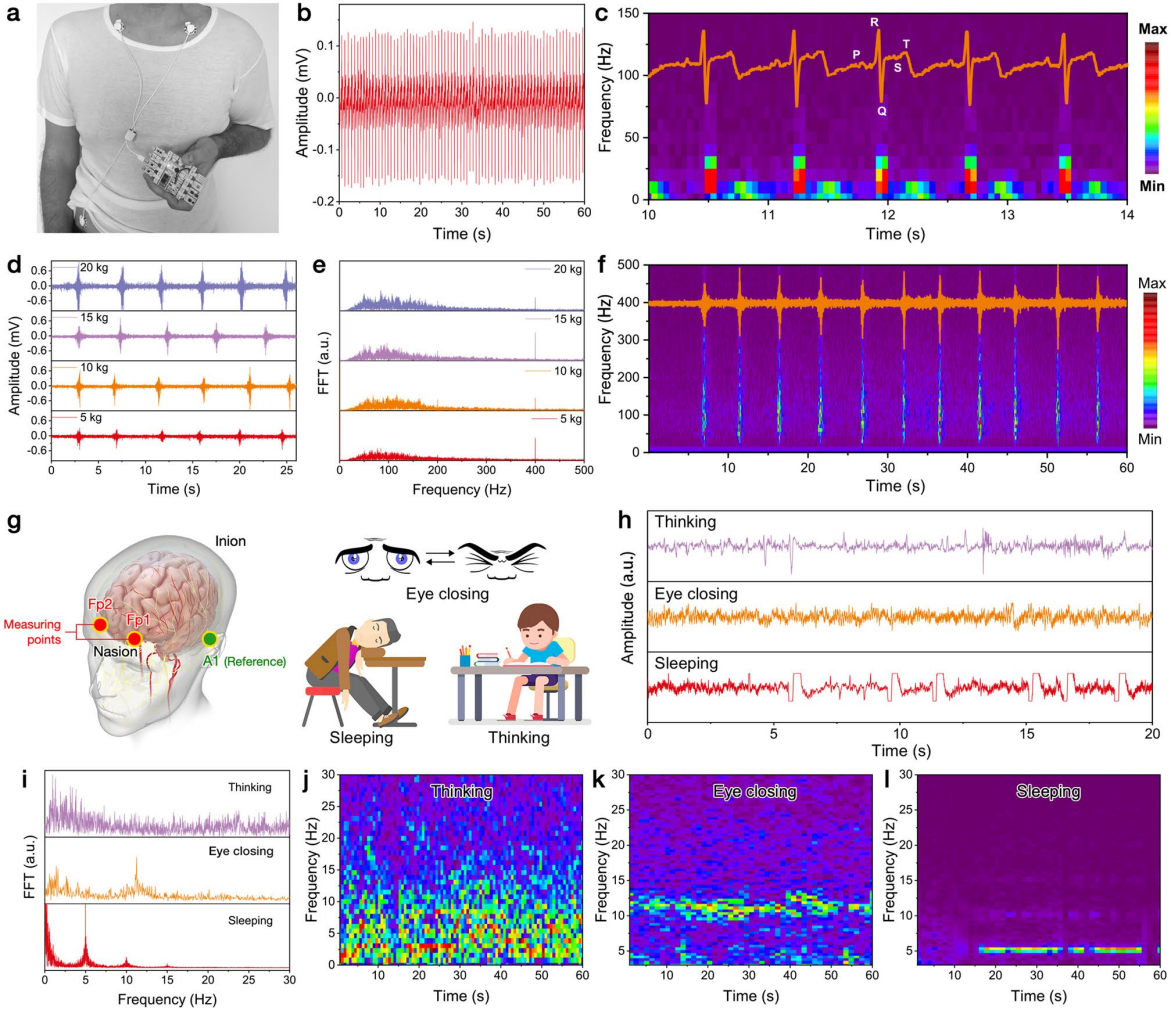
**a, b** ECG signals collected from a volunteer using the metafabric as on-skin bioelectrodes. **c** Frequency–time analysis of the ECG signals. **d** sEMG signals collected from different gripping forces, and **e** corresponding FFT analysis. **f** Frequency–time analysis of the sEMG signals (gripping force: 15 kg). **g** Schematic diagram of the EEG measurement position and mental states for EEG monitoring. **h** Recorded EEG signals using the metafabric as electrophysiological sensors. **i** FFT-processed frequency distributions of the EEG signals. **j**–**l** Time–frequency spectrograms of the EEG signals within beta band (5–30 Hz) under three mental states, showing significantly higher signal intensities during thinking

The ultra-elasticity, skin-like Young’s modulus, excellent breathability, and deformable conductivity make our PDRC metafabric promising on-skin electronic to collect various human electrophysiological signals including ECG, sEMG, and EEG signals [42]. Accurate monitoring of the heart's biopotential activities could facilitate the early diagnosis of certain heart diseases like cardiac arrhythmia, inadequate coronary artery blood flow, and cardiac ischemia [43]. Three pieces of metafabric electrodes were directly used as skin-attachable bioelectrodes, which were mounted onto the left/right forearms and left ankle of a male volunteer as shown in Fig. 5a. Clearly, high-fidelity and repeatable *P*-wave, QRS complex, and *T*-wave corresponding to the cardiac activities of atrial depolarization, ventricular depolarization, and ventricular repolarization can be found in the ECG waveforms (Fig. 5b, c). More importantly, the ECG signals acquired by the metafabric electrodes can be further used to determine the frequency identification of P-QRS-T peaks. As shown in the frequency–time plot in Fig. 5c, the amplitude–time curve is perfectly coordinated with the frequency spectrum, providing precious early detection of potential heart diseases.

In addition, sEMG signals corresponding to muscle activities were also monitored by attaching the metafabric bioelectrodes on the forearm (detection electrodes) and the elbow joint (reference electrodes). To start with, the sEMG signals of different gripping forces were monitored. As depicted in Fig. 5d, through repetitive gripping of 5–20 kg forces on the gripper, stable and consistent biopotential signals from the muscle contractions can be recorded under the same gripping force. The signal-to-noise ratio of the sEMG signals is calculated as ~ 18.3 dB based on Eq. (4):

4 $${\text{SNR}} \left( {{\text{dB}}} \right) = 20 \times \log_{10} \frac{{\sqrt {\mathop \sum \nolimits_{k = 1}^{N} V_{{{\text{signal}}\left( k \right)}}^{2} } }}{{\sqrt {\mathop \sum \nolimits_{k = 1}^{N} V_{{{\text{noise}}\left( k \right)}}^{2} } }}$$

where *N* is the number of samples, and *V*_signal(k)_ and *V*_noise(k)_ are the voltage values of the signal and noise, respectively. Time domain and frequency domain analysis was conducted through Fourier transform to better understand the muscle activities. Spectral analysis and amplitude assessments are frequently carried out to reveal the physiological and bio-mechanical processes that exist in the muscles. For example, the amplitude attribute is usually applied as a qualitative indication of muscle state [44]. As presented in Fig. 5e, f, the general frequency range of muscle activities is around 0 ~ 500 Hz, with major ranges of 50 ~ 200 Hz. Meanwhile, it can be observed that the increase in gripping force leads to the broadening of frequency distribution, indicating more muscles contracted during the gripping process. Additionally, the metafabric electrodes can monitor the steadily increased signal intensity when progressively increasing the gripping force, proving the unlimited potential of the metafabric in the area of sports management.

Aside from the ECG and sEMG signals, reliable detection of cerebral activities via high-precision, point-of-care EEG signal can provide valuable information on many neurological disorders and brain-related diseases and is of crucial importance for studying cognitive behaviors [45, 46]. Cerebral activities of humans can be classified into five frequency bands, including *γ* wave (> 40 Hz), *β* wave (12–40 Hz), *α* wave (8–12 Hz), *θ* wave (4–8 Hz), and *δ* wave (0–4 Hz) [47]. Each wave band corresponds to specific brain activity. For instance, low-frequency *θ* waves are frequently observed during deep sleeping, whereas high-frequency *β* waves can be found during attentional mental states. The metafabric electrodes can be easily applied to collect precise EEG signals by attaching them on the left (Fp1) and right (Fp2) sides of the forehead as recording electrodes, and a reference electrode was mounted on the left occipital region (A1), following the generally-accepted 10–20 system of EEG electrodes placement (Fig. 5g) [46]. Three typical mental states of the volunteer were, respectively, recorded to assess the performance of the metafabrics as EEG electrodes. Signals of “closed eyes” were recorded in a conscious and clear state. Signals corresponding to “thinking” were obtained when the volunteer was solving a complex math calculation. EEG signals of “sleeping” were collected when the volunteer was lying and sleeping (Fig. 5h). As depicted in Fig. 5i, the collected EEG signals indicate energetic and high-frequency cerebral activities when performing a mathematical calculation, in contrast to the prominent low-frequency neural activities observed during eye closing and deep sleeping. Furthermore, Fast Fourier transform (FFT) was utilized to analyze the EEG signals (Fig. 5j), which show significant *α* waves (~ 13 Hz) during eye closing, *θ* waves (~ 5 Hz) during deep sleeping, and *β* waves (14–30 Hz) during thinking. Additionally, the time–frequency spectrograms of the recorded EEG signals show remarkable differences in signal intensities for the three mental states (Fig. 5k), confirming the accuracy and high resolution of the metafabric bioelectrodes.

## Conclusions

In this work, a health monitoring metafabric with exceptional passive cooling capability is successfully developed for comfort-wearing skin electronics. Owing to the outstanding UV–Vis–NIR sunlight reflection, high MIR bands emissivity as well as the thermal-fused porous fiber networks, developed metafabric achieves a maximum cooling effect up to 17, 10, and 12 °C compared to casting SEBS film, nylon fabric, and simulated skin, respectively. The PDRC metafabric printed with a liquid metal circuit is also used to collect high-fidelity electrophysiological signals including ECG, sEMG, and EEG, which is especially helpful in sports management and early diagnosis of cardiovascular and cerebrovascular diseases. Thus, the metafabric with multifunctionalities including skin-conformable health monitoring and electricity-free cooling shows broad prospects in low-carbon wearable electronics.

### Supplementary Information

Below is the link to the electronic supplementary material.

Supplementary file1 (PDF 922 KB)
