# Supplementary material for: Superelastic Radiative Cooling Metafabric for Comfortable Epidermal Electrophysiological Monitoring
Source: Nanomicro Lett. 2023 Jul 13;15:181. doi: 10.1007/s40820-023-01156-9 (PMC10344855; doi:10.1007/s40820-023-01156-9)
Supplement: Supplementary file 1 — Supplementary file1 (PDF 922 KB) [file 40820_2023_1156_MOESM1_ESM.pdf]

Supporting Information for

Superelastic Radiative Cooling Metafabric for Comfortable Epidermal Electrophysiological Monitoring

Jiancheng Dong[1], Yidong Peng[1], Yiting Zhang[1], Yujia Chai[1], Jiayan Long[1], Yuxi Zhang[1], Yan Zhao[2], Yunpeng Huang[1,] * and Tianxi Liu[1,] *

[1] Key Laboratory of Synthetic and Biological Colloids, Ministry of Education, School of Chemical and Material Engineering, Jiangnan University, Wuxi, 214122, P. R. China

[2] College of Energy Material and Chemistry, College of Chemistry and Chemical Engineering, Inner Mongolia University, Hohhot, 010021, P. R. China

*Corresponding authors. E-mail: hypjnu@jiangnan.edu.cn (Y. P. Huang), txliu@jiangnan.edu.cn (T. X. Liu)

Supplementary Figures

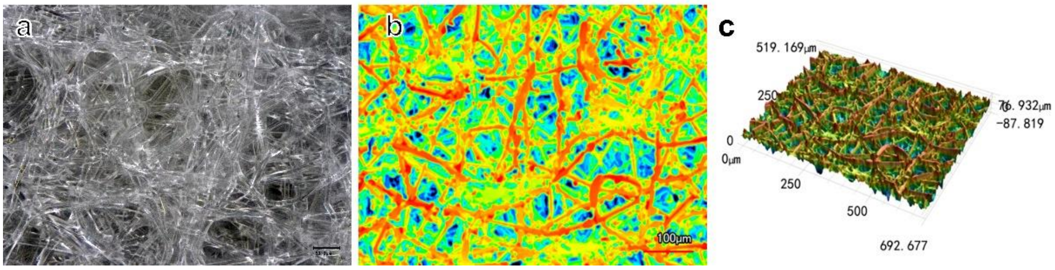

Fig. S1 (**a**) Optical microscopic, (**b**) contour map and (**c**) 3D surface topographic images of pure SEBS fabric

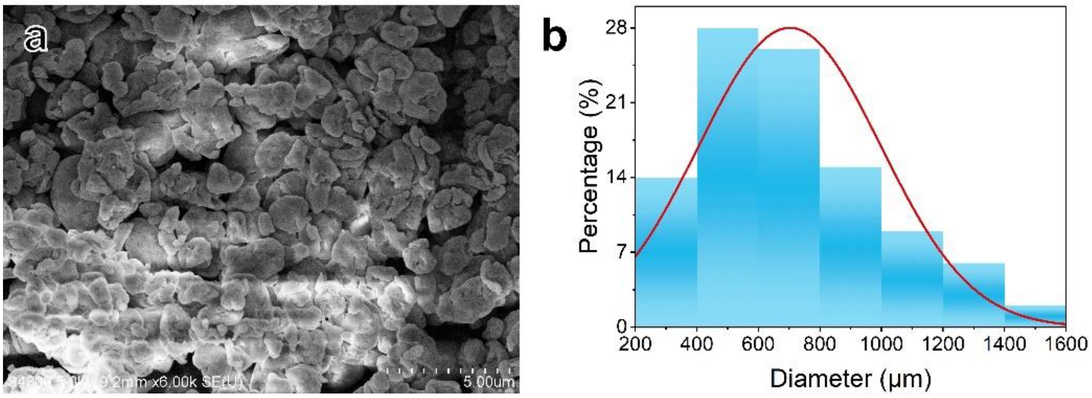

Fig. S2 (**a**) SEM image and (**b**) diameter distribution of PTFE microparticles applied in this study

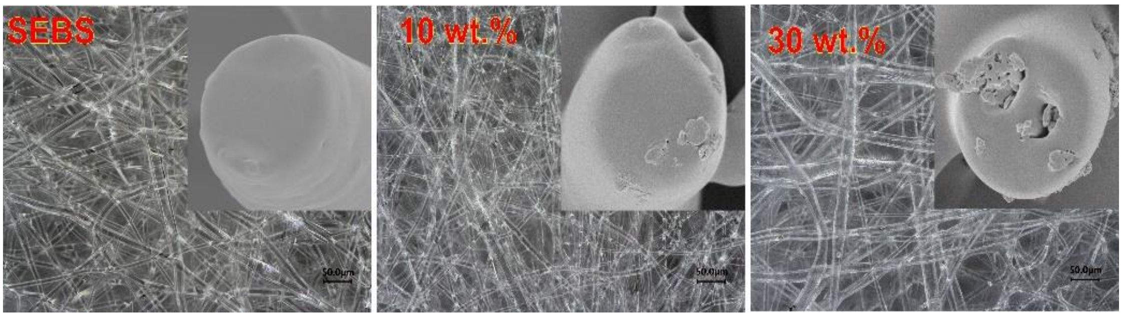

Fig. S3 Optical and SEM images of pure SEBS and SPM microfibers with 10 wt% and 30 wt% PTFE loading

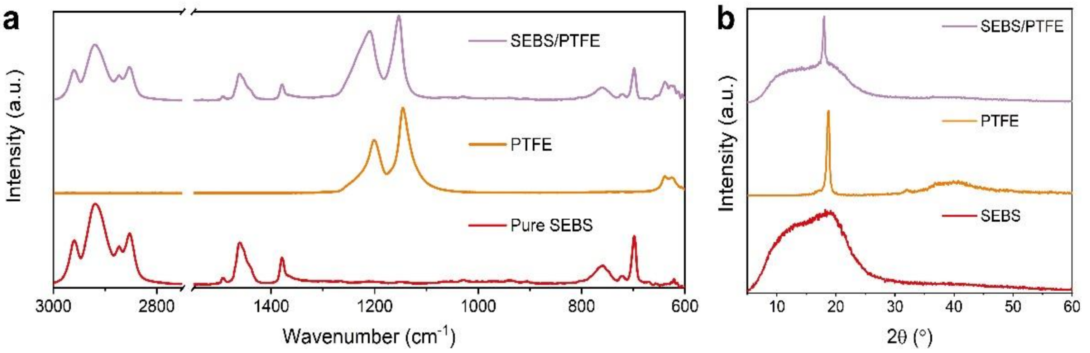

Fig. S4 (**a**) FTIR spectra and (**b**) XRD patterns of pure SEBS fabric, PTFE microparticles and SPM fabric

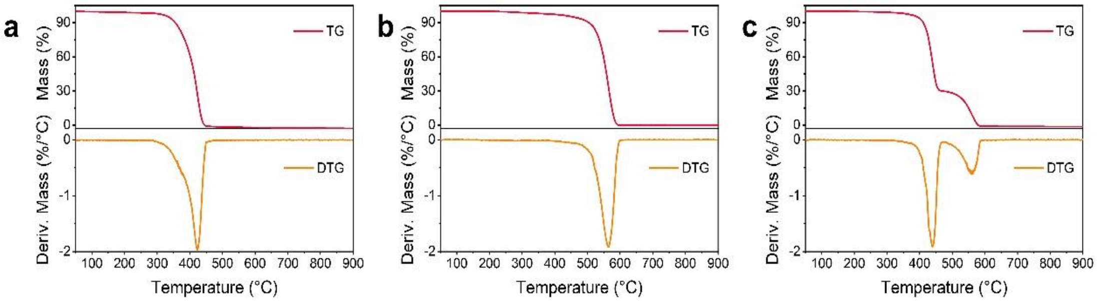

Fig. S5 TGA curves of (**a**) SEBS, (**b**) PTFE and (**c**) SPM microfibers

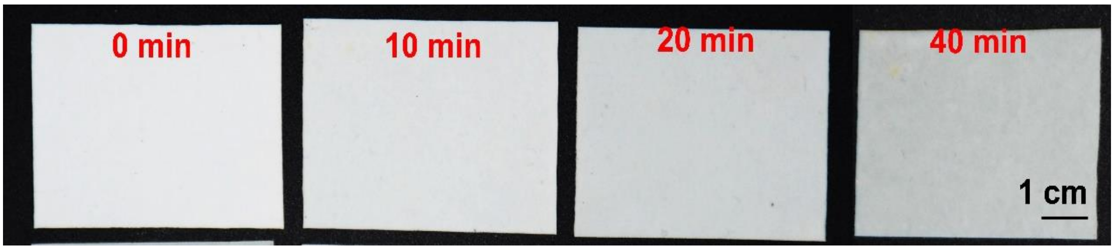

Fig. S6 Digital photos of tSPM fabrics treated for different times under 150 °C

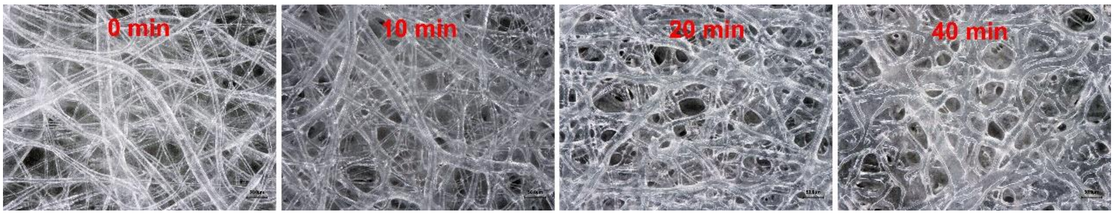

Fig. S7 Optical microscopic images of tSPM fabrics treated for different times under 150 °C

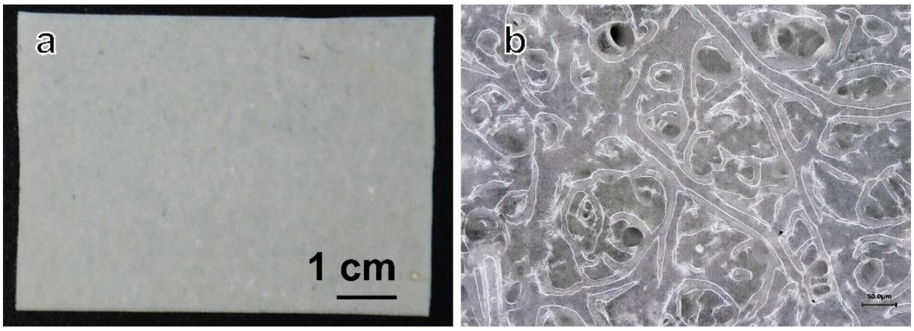

Fig. S8 (**a**) Digital and (**b**) optical images of tSPM fabric after thermal treatment for 60 min

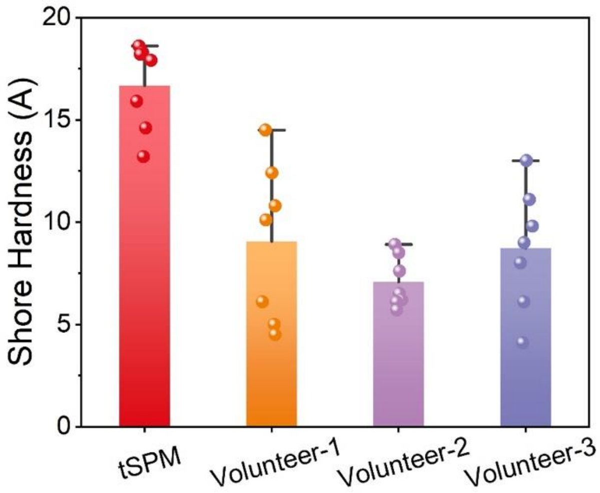

Fig. S9 Shore hardness of tSPM fabric, and skins of volunteer-1 (male, 28 years old), volunteer-2 (male, 24 years old) and volunteer-3 (female, 26 years old)

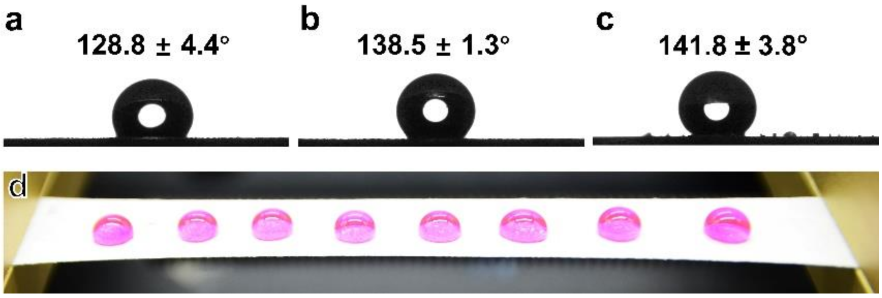

Fig. S10 Water contact angles of (**a**) pure SEBS fabric, (**b**) tSPM fabric, (**c**) PTFE particles, and (**d**) digital photo of water droplets on tSPM fabric under a strain of 1000%

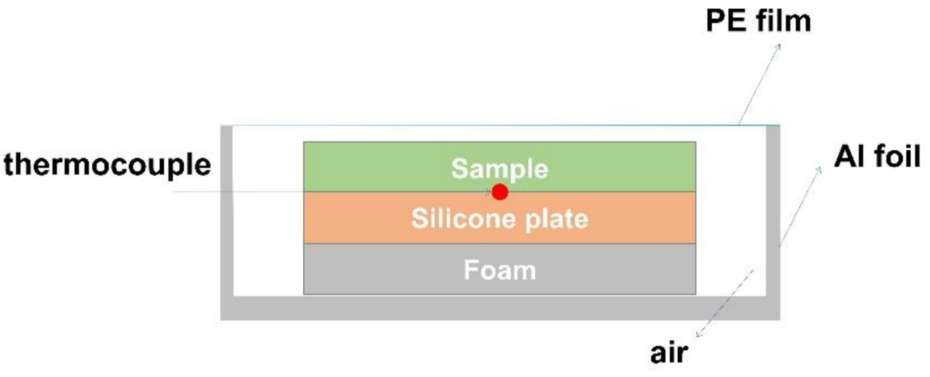

Fig. S11 Schematic of the set-up for radiative cooling tests

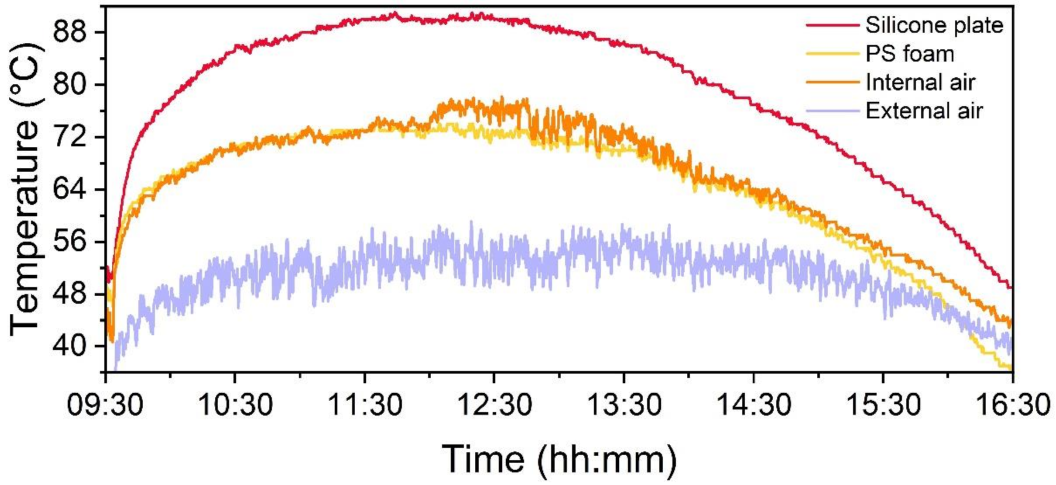

Fig. S12 Temperatures of silicone plate, PS foam and air inside/outside the PS box under direct sunlight irradiation

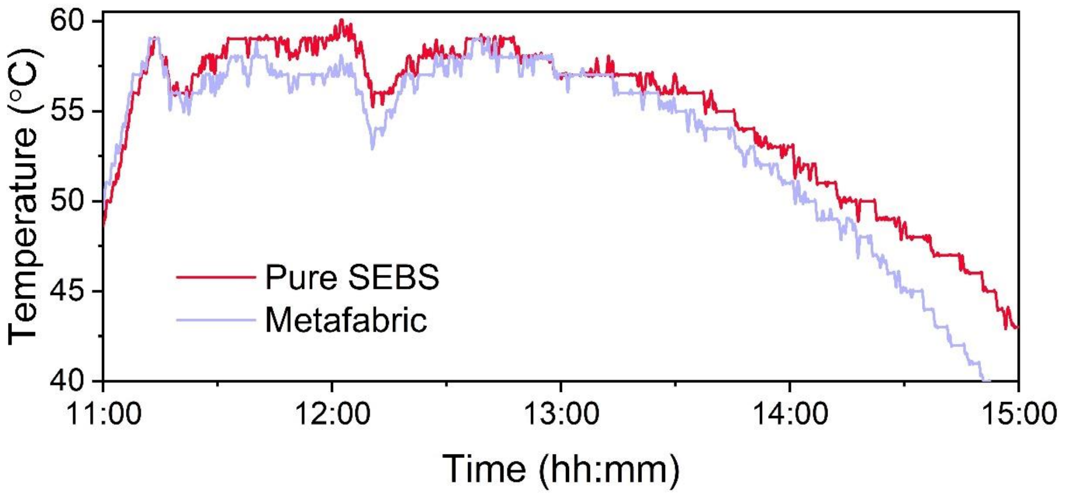

Fig. S13 Temperature comparison of pure SEBS microfibers and metafabric under passive cooling tests
